# Can cross-sector governance improve urban health resource supply? Evidence from China's Healthy City pilot policy

**DOI:** 10.3389/fpubh.2026.1812673

**Published:** 2026-06-08

**Authors:** Jiexun Zheng

**Affiliations:** Law School of Fuzhou University, Fuzhou, China

**Keywords:** China, cross-sector governance, difference-in-differences, health in all policies, health resource allocation, healthy city, policy evaluation, urban health

## Abstract

**Background:**

China launched the national Healthy City pilot program in 2016 to translate Health in All Policies into municipal governance. The policy differs from sector-specific health interventions because it embeds health goals into urban planning, fiscal allocation, interdepartmental coordination, and accountability routines. Whether this cross-sector governance approach increases urban health resource supply remains empirically unresolved.

**Methods:**

This study constructs a city-year panel of 293 prefecture-level cities from 2007 to 2022. It applies a two-way fixed effects difference-in-differences (DID) design to estimate the effect of the Healthy City pilot on three health resource outcomes: hospitals and health centers, hospital beds, and licensed physicians. Event-study estimates, per-capita specifications with socioeconomic controls, six robustness checks, and a placebo test assess the credibility of the estimates.

**Results:**

The baseline DID estimates show that the pilot increased the log number of hospitals and health centers by 0.248 (SE = 0.061, *p* < 0.001), beds by 0.089 (SE = 0.033, *p* = 0.007), and physicians by 0.072 (SE = 0.030, *p* = 0.015). Event-study estimates show no differential pre-policy trends and indicate that effects accumulated after implementation. The results remain positive across per-capita and robustness specifications. Heterogeneity analysis shows larger gains in western and central cities, cities with lower baseline resource endowments, and cities with weaker fiscal capacity.

**Conclusions:**

Cross-sector governance can expand urban health resource supply by aligning fiscal priorities and administrative coordination around health goals. The largest effect appears for institutional capacity, while the smaller physician effect suggests that workforce supply requires complementary policies. Resource-scarce cities gain more from the pilot, indicating that governance-oriented health policy can support more equitable health system strengthening.

## 1 Introduction

China's urban health resource stock expanded between 2000 and 2022, yet the distribution of hospitals, beds, and physicians across prefecture-level cities remains uneven. Recent city-level evidence documents persistent spatial disparities in these three resource categories and links healthcare resource inequality to uneven economic development across Chinese cities (1, 2). The central government has deployed multiple policy instruments to address this imbalance, ranging from medical insurance reform to hospital governance restructuring. Among these instruments, the Healthy City pilot program, launched in November 2016 under the guidance of the National Patriotic Health Campaign Committee, represents a distinct approach because it embeds health objectives into cross-sector urban governance. The program designated 32 prefecture-level cities as first-batch pilot sites and required each to implement a structured package of governance reforms, including leadership coordination mechanisms, health-oriented development planning, population-wide health management, and institutionalized evaluation and accountability (3, 4).

The Healthy City concept originates from the World Health Organization's (WHO) Healthy Cities Initiative, which promotes Health in All Policies (HiAP) as a governance strategy for addressing social determinants of health (5). In this policy context, cross-sector governance refers to municipal arrangements that incorporate health objectives into non-health sectors through intersectoral bodies, joint projects, shared goals, funding arrangements, accountability routines, and usable indicators (3, 6, 7). China's adaptation of this concept operates through a “6+X” implementation framework that combines six standardized governance modules with locally customized innovation components. The six standardized modules cover leadership coordination, health-oriented development planning, key health projects, healthy cells community programs, population-wide health management, and evaluation with accountability. The “X” component allows each pilot city to design locally tailored innovation measures based on its specific health challenges and governance capacity. This design creates variation in policy exposure across cities and over time, which provides a natural setting for causal identification.

The policy context is embedded in China's broader Healthy China 2030 strategy, which elevates population health to a national development priority. The Healthy City pilot serves as a local implementation vehicle for this national strategy, translating top-level policy objectives into city-level governance actions. The pilot selection process considered geographic balance, economic diversity, and administrative willingness, producing a treatment group that spans eastern, central, and western regions of China. This geographic spread reduces concerns about selection on a single dimension of city characteristics and strengthens the external validity of the estimated effects.

Despite the policy's prominence, empirical evidence on its health resource effects remains limited. Existing studies on China's Healthy City program focus primarily on evaluation index construction (8, 9), descriptive case analyses (10), or outcomes unrelated to health resource supply, such as industrial structure upgrading (11) and labor supply (12). Studies that do examine health outcomes tend to rely on cross-sectional comparisons or qualitative assessments, which cannot isolate the causal effect of the policy from confounding trends (13). The broader healthy cities literature, while rich in conceptual frameworks and indicator systems (14–16), provides limited city-panel causal evidence on whether governance-oriented policies produce measurable changes in health resource supply. This absence of causal evidence is particularly notable given the scale of the policy: the 32 first-batch pilot cities collectively serve over 200 million residents, making the program one of the largest urban health governance experiments in the world.

This gap matters for two reasons. First, local governments allocate fiscal resources based on expected policy returns. Without causal evidence on whether the Healthy City pilot increases health resource supply, investment decisions remain uninformed. City officials face uncertainty about whether the governance reforms mandated by the pilot program produce tangible health infrastructure improvements or primarily generate administrative compliance without material resource changes. Second, the HiAP approach represents a governance model that differs from direct health system interventions. Establishing whether this model produces tangible resource outcomes informs the design of future cross-sector health governance policies, both in China and in other countries adopting WHO Healthy Cities frameworks (17, 18). The distinction between governance-oriented and sector-specific health policies has practical consequences for policy design: if governance reforms produce resource effects comparable to direct investment programs, policymakers gain an additional instrument for health system strengthening that operates through institutional channels alongside fiscal transfers.

This study addresses the following research question: what is the causal effect of China's Healthy City pilot policy on city-level health resource supply, and through which governance channels does this effect operate? The study makes three contributions to the literature. First, it provides the first city-panel causal estimate of the Healthy City pilot's effect on health resource outcomes, using a difference-in-differences (DID) design with city and year fixed effects applied to 293 prefecture-level cities over 2007–2022. The long pre-treatment window (2007–2015) provides 9 years of data to test the parallel trends assumption, while the post-treatment window (2016–2022) captures 6 years of policy effects, sufficient to observe both immediate responses and medium-term accumulation. Second, it tests the dynamic pattern of policy effects through event-study analysis, which reveals whether effects accumulate over time as governance reforms take hold. This dynamic analysis distinguishes genuine governance effects from one-time administrative responses and provides evidence on the time horizon required for cross-sector governance reforms to produce observable resource changes. Third, it identifies fiscal health spending and governance capacity as mechanism channels, connecting the HiAP governance framework to measurable resource outcomes. This mechanism analysis moves beyond reduced-form treatment effect estimation to provide evidence on the causal pathways through which governance reform translates into health system capacity.

## 2 Literature review

### 2.1 Determinants of urban health resource allocation

Urban health resource allocation depends on economic development, fiscal capacity, population structure, and policy interventions. Recent evidence from Chinese cities shows that hospitals, beds, and physicians remain unevenly distributed across space, and that unequal economic development contributes to persistent resource gaps (1, 2). Urbanization accelerates demand for health services, yet the supply response varies across cities depending on local governance capacity and planning priorities. Rapid urbanization in China has produced spatial mismatches between population growth and health infrastructure expansion, particularly in central and western regions where fiscal constraints limit public investment (4). These structural determinants create baseline heterogeneity in health resource endowments, which conditions the potential effect of any new policy intervention.

The literature on health resource determinants identifies three primary drivers of cross-city variation. The first driver is economic capacity: wealthier cities generate more tax revenue, which enables larger public health budgets and attracts private health investment. The second driver is demographic structure: cities with older populations face higher per-capita health service demand, which creates pressure for resource expansion. The third driver is institutional capacity: cities with stronger coordination routines can translate fiscal inputs into health outputs more efficiently by reducing approval delays and planning fragmentation. The Healthy City pilot targets the third driver by restructuring governance institutions, which distinguishes it from policies that operate primarily through the first two channels. Understanding this distinction is essential for interpreting the policy's estimated effects, because governance-oriented interventions may produce different effect magnitudes and dynamic patterns than fiscal transfer programs.

The geographic distribution of health resources in China follows a well-documented east-west gradient. Eastern coastal cities, which benefited from early economic reforms and foreign investment, maintain higher per-capita health resource levels than central and western cities. This gradient has persisted despite decades of central government transfer payments and targeted investment programs. The persistence of this gradient suggests that fiscal transfers alone are insufficient to equalize health resource distribution, which motivates the search for complementary policy instruments such as governance reform.

### 2.2 Policy pilots and local governance effectiveness

China's governance system relies on pilot programs to test policy innovations before national rollout. Pilot cities receive policy mandates, implementation guidelines, and evaluation pressure from higher-level governments, which creates incentives for local officials to allocate resources toward pilot objectives (19). The pilot program mechanism operates through a combination of top-down mandates and bottom-up implementation discretion. Central authorities define the policy objectives and evaluation criteria, while local governments retain flexibility in choosing specific implementation strategies. This combination creates a principal-agent dynamic in which local officials face both performance incentives and resource constraints.

Empirical studies on other Chinese pilot programs, such as the National Civilized City campaign, demonstrate that governance competition among cities produces measurable changes in local public goods provision (19). The National Civilized City program shares structural similarities with the Healthy City pilot: both designate specific cities for governance reform, both impose evaluation and accountability requirements, and both create reputational incentives for local officials. The key difference is the policy domain: the Civilized City program targets broad urban governance quality, while the Healthy City pilot focuses specifically on health-related governance outcomes. Evidence from the Civilized City program suggests that pilot designation increases local government attention to the targeted policy domain, which translates into measurable resource allocation changes.

The effectiveness of pilot programs depends on implementation intensity, monitoring mechanisms, and the alignment between policy objectives and local government incentives. Programs with strong monitoring and clear evaluation metrics tend to produce larger effects than programs with vague objectives and weak accountability. The Healthy City pilot incorporates a structured evaluation system with quantitative indicators, which strengthens the accountability channel. Existing DID studies have used the Healthy City pilot to examine municipal solid waste management and mental health inequalities, showing that the policy can affect urban governance outcomes and health-related welfare (20, 21). These studies leave open whether the same governance reform changes the supply of health institutions, beds, and physicians, which are direct capacity inputs in the urban health system.

### 2.3 Healthy city and health in all policies frameworks

The WHO Healthy Cities Initiative promotes a governance approach in which municipal governments integrate health considerations into all policy domains (16). HiAP operationalizes this approach through cross-sector coordination, health impact assessment, and accountability mechanisms that connect urban policy decisions to the social determinants of health (5).

International Healthy Cities evaluations clarify how this governance approach works in practice. European network experience shows that network governance can strengthen participation, local policy-making, and intersectoral action, while health impact assessment helps translate HiAP into municipal decision routines (22, 23). Local HiAP evaluation further identifies intersectoral bodies, joint projects, and implementation maturity as observable conditions for moving from policy commitment to municipal action (6). These findings support the claim that Healthy Cities programs operate through governance capacity, although most international evidence remains process-oriented or associational.

China's Healthy City program adapts the WHO framework to a hierarchical administrative context with strong central-local accountability linkages. Compared with the network-based European experience, the Chinese approach relies more heavily on government-led policy tools, supply-oriented instruments, and health service construction as a policy domain (3, 6, 22). The program requires pilot cities to establish cross-departmental coordination committees, develop health-oriented urban plans, implement “healthy cells” projects at the community level, and submit to regular evaluation by national authorities. This multi-level implementation structure creates governance pressure at multiple administrative tiers simultaneously.

Existing Chinese studies on this program have constructed evaluation index systems (8, 9), analyzed policy document content (24), and described implementation processes (13). Recent causal evaluations have examined outcomes such as municipal solid waste management and mental health inequalities (20, 21). This evidence shows that the pilot can alter urban governance and health-related welfare, while leaving unresolved whether the same governance reform changes the supply of health institutions, beds, and physicians. The resource-supply question requires city-panel causal evidence because health resource growth can reflect pre-existing local capacity, common national trends, or the pilot policy itself.

### 2.4 Methodological considerations in healthy city evaluation

Evaluating the causal effect of healthy city policies presents methodological challenges. Observational studies face selection bias because pilot cities may differ systematically from non-pilot cities in ways that correlate with health resource trajectories (25). If pilot cities were selected because they already had stronger health governance or faster resource growth, naive comparisons between pilot and non-pilot cities would overestimate the policy effect. Cross-sectional comparisons cannot distinguish policy effects from pre-existing trends, and before-after comparisons within pilot cities cannot separate the policy effect from common temporal shocks that affect all cities simultaneously.

The DID design addresses these concerns by comparing changes in outcomes between treated and control cities before and after policy implementation, under the assumption that both groups would have followed parallel trends in the absence of treatment (26). The parallel trends assumption is the key identifying assumption: it requires that, absent the policy, pilot and non-pilot cities would have experienced the same changes in health resource outcomes over time. This assumption does not require that the two groups have identical outcome levels, only that their outcome trajectories would have been parallel. The TWFE specification absorbs time-invariant city characteristics through city fixed effects and common temporal shocks through year fixed effects, isolating the differential change attributable to the policy.

Event-study extensions of the DID framework provide a direct test of this parallel trends assumption and reveal the dynamic pattern of policy effects (27). By estimating separate coefficients for each event-time period relative to the policy year, the event-study design allows visual and statistical inspection of pre-treatment coefficient patterns. If the parallel trends assumption holds, pre-treatment coefficients should be statistically indistinguishable from zero. A joint *F*-test of the null hypothesis that all pre-treatment coefficients equal zero provides a formal statistical test of this assumption. Post-treatment coefficients trace the dynamic evolution of the policy effect, revealing whether effects are immediate or accumulating, temporary or persistent.

This study adopts both approaches and supplements them with placebo tests and multiple robustness specifications to strengthen causal identification. The combination of baseline DID, event-study analysis, placebo permutation tests, and six alternative specifications provides a comprehensive identification strategy that addresses the primary threats to causal inference in this setting. The placebo test provides a nonparametric benchmark by simulating the distribution of DID coefficients under random treatment assignment, which allows assessment of whether the true estimate is statistically distinguishable from chance. The robustness specifications address specific threats including province-level confounders, differential pre-existing trends, outlier influence, structural differences between municipalities and prefecture-level cities, and unbalanced panel composition.

## 3 Theoretical framework and hypotheses

### 3.1 Policy mechanism: from governance reform to resource expansion

The Healthy City pilot operates through a governance mechanism that differs from direct health system interventions. Traditional health resource policies, such as hospital construction subsidies or physician training programs, target the health sector directly. The Healthy City pilot instead restructures local governance by requiring cross-departmental coordination, health-oriented planning integration, and performance accountability. Local HiAP evaluation identifies intersectoral bodies and joint projects as markers of mature implementation, while reviews of multisectoral health policy identify political leadership, shared goals, funding, accountability, and indicators as recurring implementation conditions (6, 7). China's Healthy City policy also gives health service construction a concrete place within the policy tool system (3). This governance restructuring affects health resource supply through two channels.

The first channel operates through fiscal reallocation. The pilot program requires cities to prioritize health in fiscal planning and to increase the share of public expenditure directed toward health infrastructure and services. Cross-departmental coordination committees, mandated by the program, create institutional pressure for budget offices to allocate resources toward health objectives that would otherwise compete with other spending priorities (28). In the Chinese fiscal system, local governments exercise substantial discretion over expenditure allocation within broad central government guidelines. Health spending competes with infrastructure, education, and economic development for limited fiscal resources. The Healthy City pilot's governance requirements shift the relative priority of health spending by creating evaluation metrics that directly measure health resource outcomes. Local officials who fail to meet these metrics face reputational costs in the national assessment system, which creates incentives to redirect fiscal resources toward health objectives. This fiscal channel produces direct increases in health resource inputs, including hospital construction, bed expansion, and physician recruitment.

The magnitude of the fiscal channel effect depends on the city's initial fiscal capacity and the degree of competition between health and non-health spending priorities. Cities with tighter fiscal constraints face stronger trade-offs between health spending and other priorities, which means that the governance pressure created by the pilot program produces a larger marginal reallocation effect. Cities with abundant fiscal resources may already allocate sufficient funds to health, reducing the marginal impact of governance-induced reallocation. This theoretical prediction generates a testable heterogeneity hypothesis: the policy effect should be larger in cities with lower fiscal capacity.

The second channel operates through governance capacity improvement. The program's evaluation and accountability requirements create performance incentives for local officials. Cities that fail to meet health development targets face reputational costs in the national assessment system. This accountability pressure motivates local governments to improve administrative efficiency in health resource planning, streamline approval processes for health facility construction, and strengthen coordination between health bureaus and other government departments (29). The governance capacity channel operates through reduced bureaucratic friction: in the absence of cross-departmental coordination, health facility construction proposals may face delays in land use approval, environmental review, and construction permitting, each of which involves a different government department. The Healthy City pilot's coordination committee structure reduces these inter-departmental delays by creating a single institutional venue for resolving cross-cutting issues. Governance capacity improvements reduce bureaucratic friction in resource allocation, which accelerates the translation of fiscal inputs into actual health resource outputs.

The two channels are complementary. Fiscal reallocation supplies the material inputs for health resource expansion, and governance capacity reduces the bureaucratic bottlenecks that slow facility approval, budget execution, and interdepartmental coordination. The Healthy City pilot activates both channels simultaneously, so the policy's total effect should reflect their joint operation. This complementarity implies that mechanism regressions should find positive policy effects on fiscal health spending intensity and governance capacity, and positive associations between those channels and health resource outcomes.

### 3.2 Hypotheses

Based on this theoretical framework, the study tests three hypotheses.

HiAP maturity implies that health goals become resource-relevant when municipal governments convert broad commitments into intersectoral bodies, joint projects, funding arrangements, accountability routines, and indicators (6, 7). In the Chinese Healthy City pilot, these arrangements give local officials a reason to prioritize health service construction and a coordination venue for resolving land use, budget, planning, and implementation constraints (3). The expected level effect applies to city-level resource supply because institutions, beds, and physicians are the main administrative outputs through which local governments can expand health system capacity.

H1 (Level effect): The Healthy City pilot increases total health resource supply in pilot cities relative to non-pilot cities.

The magnitude of this level effect should vary across resource dimensions because institutions, beds, and physicians adjust at different speeds. Hospitals and health centers can respond through facility approval, registration, and construction, while bed expansion depends on facility investment and renovation, and physician growth depends on labor-market and training pipelines. This boundary condition implies a stronger short- to medium-term effect on institutional capacity and a smaller effect on human resource capacity.

Governance reforms require time to produce observable resource changes. Intersectoral bodies must be established, shared goals must enter local planning, funding must be allocated, accountability routines must become salient to officials, and construction or staffing decisions must move through administrative procedures. The policy effect should therefore strengthen over time as HiAP implementation matures and capital investments reach completion.

H2 (Dynamic effect): The policy effect strengthens over time after pilot implementation.

This dynamic warrant also defines the event-study expectation. A governance-driven effect should not appear as a large pre-policy divergence or a 1-year reporting jump at implementation. It should appear as statistically similar pre-policy trends followed by accumulating post-policy coefficients as planning, funding, and coordination begin to affect resource supply.

The fiscal and governance channels specify how the level and dynamic effects should occur. If the pilot works through cross-sector governance, treated cities should increase fiscal health spending intensity and improve measured governance capacity. These channel variables should also be positively associated with health resource outcomes because fiscal priority supplies material inputs, while governance capacity reduces coordination costs and implementation delays.

H3 (Governance channel): Cross-sector coordination and assessment pressure improve policy execution, leading to measurable resource growth through fiscal and governance channels.

The mechanism test is bounded by the available data. It can show whether the pilot is associated with the proposed channel variables and whether those channels predict resource outcomes. It does not establish a complete structural mediation model, so the mechanism results are interpreted as channel evidence that is consistent with the theoretical pathway.

## 4 Data, variables, and empirical strategy

### 4.1 Data sources

This study draws on three data sources. Health resource data are from the China Health Statistical Yearbook. This source provides annual city-level counts of hospitals and health centers, beds, and physicians. These variables measure the formal supply capacity of the urban health system and serve as the main outcome variables.

Pilot policy information is based on the National Patriotic Health Campaign Committee Office's *Notice on Carrying Out Healthy City Pilot Work* [Document No. 4 [2016]]. The policy treatment year is 2016, corresponding to the approval of the first batch of 32 pilot cities in November 2016. The treatment indicator (*HC*_*i*_) equals one for those pilot cities and zero otherwise. The post-policy indicator (*Post*_*t*_) equals one from 2016 onward. The DID interaction term (*DID*_*it*_ = *HC*_*i*_×*Post*_*t*_) captures the differential post-policy change in treated cities.

City-level socioeconomic controls are from the China City Statistical Yearbook. This source provides GDP per capita, secondary and tertiary industry shares, fiscal expenditure per capita, and average population. These variables capture economic development, industrial structure, fiscal capacity, and population scale, which are primary determinants of health resource allocation at the city level. The control panel covers 2007 to 2018 in the controlled specifications.

### 4.2 Data cleaning and sample construction

The analytical panel is constructed at the city-year level. The health resource data, DID policy indicators, and city socioeconomic panel are merged by city code and year. After merging, duplicate city-year records are removed before analysis so that each retained observation represents one city in one calendar year. Observations with missing variables are excluded from the corresponding regression by listwise deletion. No imputation is applied; the retained sample is therefore defined by the variables used in each regression. This rule means that the estimation sample can differ across outcomes and specifications depending on which outcome and control variables are required. Sample A covers 293 prefecture-level cities from 2007 to 2022 and uses the full post-treatment window without socioeconomic controls, yielding 4,588 to 4,596 city-year observations depending on the outcome variable. Sample B covers 293 cities from 2007 to 2018 and includes socioeconomic controls, yielding 2,540 city-year observations in the controlled regressions. Of the 293 cities, 32 are treated pilot cities and 261 serve as controls.

### 4.3 Variables

Table 1 defines the variables, units, and data sources used in the empirical analysis. The three primary outcome variables are hospitals and health centers, beds, and physicians. Hospitals and health centers are measured as a count of institutions. Beds are measured as a count of beds. Physicians are measured as a count of licensed physicians. The main regressions use the natural logarithm of these counts: ln_*hosp*, ln_*beds*, and ln_*doctors*. The logarithmic transformation reduces the influence of extreme values and allows coefficients to be interpreted as approximate percentage changes. Specifically, a DID coefficient of β on the log outcome implies an approximate 100 × (*e*^β^−1)% change in the level outcome. For Sample B, the study also constructs per-capita versions of these outcomes, dividing by average population and scaling to per 10,000 population before taking logarithms. The per-capita transformation accounts for differences in city population size and ensures that the estimated effects reflect changes in resource intensity.

**Table 1:** Variable definitions, units, and sources.

Variable	Definition	Unit	Source
*HC*_*i*_	Healthy City pilot city indicator	0/1	Policy notice
*Post*_*t*_	Post-2016 period indicator	0/1	Policy notice
*DID*_*it*_	*HC*_*i*_×*Post*_*t*_	0/1	Constructed
Hospitals and health centers	Health service institutions	count	China Health Statistical Yearbook
Beds	Hospital beds	count	China Health Statistical Yearbook
Physicians	Licensed physicians	count	China Health Statistical Yearbook
Hospitals per 10k	Institutions per 10,000 population	per 10,000 population	Constructed
Beds per 10k	Beds per 10,000 population	per 10,000 population	Constructed
Physicians per 10k	Physicians per 10,000 population	per 10,000 population	Constructed
GDP per capita	City economic development	yuan per person	China City Statistical Yearbook
Secondary industry share	Secondary industry share of GDP	percent	China City Statistical Yearbook
Tertiary industry share	Tertiary industry share of GDP	percent	China City Statistical Yearbook
Fiscal expenditure per capita	Local fiscal spending capacity	yuan per person	China City Statistical Yearbook

Log specifications use ln(1+*x*). Per-capita outcomes are scaled to per 10,000 population before log transformation.

The three outcome variables capture different dimensions of health resource supply. The number of hospitals and health centers measures institutional capacity, reflecting the availability of health service delivery points within a city. The number of beds measures physical infrastructure capacity, reflecting the ability to provide inpatient care. The number of physicians measures human resource capacity, reflecting the availability of trained medical professionals. These three dimensions are complementary: a city needs institutions to house services, beds to accommodate patients, and physicians to deliver care. The policy may affect these dimensions differently because they involve different production processes and adjustment speeds.

Control variables in Sample B include the log of GDP per capita (ln_*pgdp*), the secondary industry share of GDP (*ind*2_*share*), the tertiary industry share of GDP (*ind*3_*share*), and the log of fiscal expenditure per capita (ln_*fiscal*_*exp*_*pc*). These variables capture economic development, industrial structure, and fiscal capacity, which are the primary confounders of health resource allocation at the city level. GDP per capita proxies for the overall economic capacity of the city, which affects both the demand for and supply of health services. Industrial structure shares capture the composition of the local economy: cities with larger tertiary sectors tend to have higher demand for health services and greater fiscal capacity to fund health infrastructure. Fiscal expenditure per capita measures the local government's spending capacity, which directly constrains public health investment. Including these controls in Sample B ensures that the estimated DID effect is not confounded by differential economic trends between pilot and non-pilot cities.

### 4.4 Empirical strategy

#### 4.4.1 Baseline DID

Equation 1 specifies the baseline difference-in-differences model used to estimate the average policy effect. The baseline specification estimates the average treatment effect of the Healthy City pilot using a two-way fixed effects (TWFE) model:


$$\begin{array}{l} Y_{it}=\beta \cdot DID_{it}+\gamma_{i}+\delta_{t}+\theta X_{it}+\varepsilon_{it} \end{array}$$ (1)

where *Y*_*it*_ is the health resource outcome for city *i* in year *t*, *DID*_*it*_ is the interaction of the treatment group indicator and the post-policy indicator, γ_*i*_ represents city fixed effects that absorb time-invariant city characteristics, δ_*t*_ represents year fixed effects that absorb common temporal shocks, *X*_*it*_ is a vector of time-varying controls (included in Sample B specifications), and ε_*it*_ is the error term. Standard errors are clustered at the city level to account for serial correlation within cities. The coefficient β captures the average effect of the Healthy City pilot on the outcome variable.

The DID design relies on four identifying assumptions. First, treated and control cities would have followed parallel trends in health resource outcomes in the absence of the pilot. Second, cities did not anticipate pilot designation in ways that changed resource investment before 2016. Third, the composition of cities is stable enough that estimated changes are not driven by systematic entry, exit, or boundary changes in the city panel. Fourth, no simultaneous unobserved policy shocks differentially targeted the treated cities at the same time and affected the same health resource outcomes. City fixed effects, year fixed effects, socioeconomic controls in Sample B, event-study estimates, and robustness specifications are used to assess these assumptions and reduce the main threats to identification.

#### 4.4.2 Event-study design

To test the parallel trends assumption and examine the dynamic pattern of policy effects (H2), the study estimates an event-study specification:


$$\begin{array}{l} Y_{it}=\underset{k\neq -1}{\sum }\beta_{k}\left(HC_{i}\times 1\left[t-2016=k\right]\right)+\gamma_{i}+\delta_{t}+\varepsilon_{it} \end{array}$$ (2)

where *k* indexes event time relative to the policy year (2016), and *k* = −1 serves as the reference period. The coefficients β_*k*_ for *k* < 0 test the parallel trends and no-anticipation assumptions: statistically insignificant pre-treatment coefficients indicate that treated and control cities followed similar trajectories before the policy and did not show anticipatory resource changes. The coefficients β_*k*_ for *k*≥0 trace the dynamic treatment effect after implementation. The study estimates event-time coefficients from *k* = −6 to *k* = +6 and reports joint *F*-test *p*-values for the null hypothesis that all pre-treatment coefficients equal zero.

#### 4.4.3 Robustness and placebo tests

The study implements six robustness specifications: (1) the baseline model, (2) province-by-year fixed effects to absorb province-specific temporal shocks, (3) city-specific linear time trends to control for differential pre-existing growth trajectories, (4) winsorization of outcome variables at the 1st and 99th percentiles, (5) exclusion of the four municipalities (Beijing, Shanghai, Tianjin, Chongqing) that differ structurally from prefecture-level cities, and (6) restriction to a balanced panel of cities observed in all years. A placebo test randomly reassigns treatment status to 32 non-pilot cities 500 times and re-estimates the DID coefficient for each permutation. The distribution of placebo coefficients provides a nonparametric benchmark against which to evaluate the true estimate.

## 5 Results

### 5.1 Summary statistics

Table 2 reports descriptive statistics for the two analytical samples. In Sample A (2007–2022), the mean log number of hospitals is 4.865 (SD = 0.766), the mean log number of beds is 9.525 (SD = 0.744), and the mean log number of physicians is 8.867 (SD = 0.758). These values correspond to median levels of approximately 137 hospitals, 14,000 beds, and 7,100 physicians per city, reflecting the substantial variation in health resource endowments across Chinese prefecture-level cities. The standard deviations of approximately 0.75 log points indicate that the interquartile range spans roughly a factor of two in resource levels, from smaller western cities to larger eastern cities. The treatment group indicator (*HC*) has a mean of 0.109, reflecting the 32 pilot cities among 293 total cities. The DID interaction term has a mean of 0.041, indicating that treated city-years in the post-policy period constitute approximately 4.1% of the sample. This treatment intensity is typical for Chinese pilot programs and provides sufficient variation for DID estimation while maintaining a large control group for comparison.

**Table 2:** Summary statistics.

Variable	*N*	Mean	SD	Min	P25	Median	P75	Max
Panel A: Full sample (2007–2022)								
ln(Hospitals)	4,588	4.865	0.766	1.792	4.369	4.920	5.407	8.024
ln(Beds)	4,588	9.525	0.744	6.667	9.073	9.544	10.005	11.848
ln(Doctors)	4,596	8.867	0.758	6.068	8.374	8.866	9.359	11.297
Treatment (*HC*)	4,679	0.109	0.312	0	0	0	0	1
15.6-2.4,-1499pt*HC*×*Post*	4,679	0.041	0.198	0	0	0	0	1
Panel B: Controlled sample (2007–2018)								
ln(Hosp. per 10k)	2,832	8.338	0.629	6.203	7.997	8.327	8.624	11.652
ln(Beds per 10k)	2,832	12.894	0.375	11.574	12.648	12.897	13.128	14.159
ln(Doctors per 10k)	2,832	12.190	0.439	10.711	11.884	12.175	12.458	13.691
ln(GDP p.c.)	3,115	10.392	0.685	4.605	9.945	10.376	10.866	13.056
Secondary ind. (%)	3,118	48.816	10.728	12.190	42.460	49.010	55.338	90.970
Tertiary ind. (%)	3,117	37.883	9.120	8.580	31.960	36.900	42.850	77.540
ln(Fiscal exp. p.c.)	2,833	17.999	0.603	16.298	17.614	18.001	18.350	20.914

Panel A covers 293 cities over 2007–2022. Panel B covers 293 cities over 2007–2018 with socioeconomic controls. All log variables use the transformation ln(1+*x*). Per-capita variables are scaled to per 10,000 population.

In Sample B (2007–2018), the per-capita outcome variables show less variation than the level variables, consistent with population-scaling reducing cross-city dispersion. The mean log GDP per capita is 10.392 (SD = 0.685), corresponding to a median GDP per capita of approximately 32,000 yuan. The mean secondary industry share is 48.8% (SD = 10.7), and the mean tertiary industry share is 37.9% (SD = 9.1). These values reflect the industrial composition of Chinese cities during this period, with secondary industry (manufacturing and construction) still accounting for the largest share of GDP in most cities. The mean log fiscal expenditure per capita is 17.999 (SD = 0.603), indicating moderate variation in local government spending capacity. These control variables capture the economic heterogeneity across Chinese prefecture-level cities during this period and serve as important confounders in the controlled DID specification.

The dispersion in health resources and fiscal capacity is consistent with documented regional imbalance in Chinese healthy city development and healthcare resource allocation (1, 2, 4). This pattern motivates the heterogeneity analysis by region, baseline resource level, and fiscal capacity.

### 5.2 Baseline DID results

Table 3 presents the baseline DID estimates for Sample A. The Healthy City pilot increases the log number of hospitals and health centers by 0.248 (SE = 0.061, *p* < 0.001), corresponding to an approximate 28.2% increase. The policy increases the log number of beds by 0.089 (SE = 0.033, *p* = 0.007), corresponding to a 9.3% increase. The policy increases the log number of physicians by 0.072 (SE = 0.030, *p* = 0.015), corresponding to a 7.5% increase. All three estimates are positive and statistically significant at the 5% level, supporting H1. The 95% confidence intervals exclude zero for all three outcomes: [0.128, 0.368] for hospitals, [0.025, 0.153] for beds, and [0.014, 0.131] for physicians. The effect magnitude follows a clear gradient: institutional expansion (28.2%) exceeds bed expansion (9.3%), which exceeds physician growth (7.5%). This gradient is consistent with the theoretical expectation that organizational capacity adjusts faster than human capital stock. Building or upgrading health facilities requires capital investment and administrative approval, both of which the governance reforms directly facilitate through cross-departmental coordination and health-oriented planning. Physician recruitment, by contrast, depends on medical education pipelines, professional licensing, and labor market conditions that operate on longer time horizons.

**Table 3:** Baseline DID estimates: effect of Healthy City pilot on health resources (Sample A, 2007–2022).

Variable	(1)	(2)	(3)
	ln(Hospitals)	ln(Beds)	ln(Doctors)
*DID*	0.2483^***^	0.0891^***^	0.0724^**^
	(0.0612)	(0.0327)	(0.0298)
95% CI	[0.128, 0.368]	[0.025, 0.153]	[0.014, 0.131]
City FE	Yes	Yes	Yes
Year FE	Yes	Yes	Yes
Controls	No	No	No
*N*	4,588	4,588	4,596
Cities	293	293	293
$R_{\text{within}}^{2}$	0.039	0.021	0.020

Standard errors clustered at the city level in parentheses. ^***^p < 0.01, ^**^p < 0.05, ^*^p < 0.10.

The within-*R*^2^ values are modest (0.020–0.039), which is expected in a TWFE specification where city and year fixed effects absorb the majority of outcome variation. The low within-*R*^2^ reflects the fact that the DID term captures only the differential change attributable to the policy, while most of the variation in health resources is driven by city-specific levels and common temporal trends that the fixed effects absorb.

Table 4 presents the per-capita DID estimates for Sample B with socioeconomic controls. The results are consistent with the baseline estimates. The policy increases the log number of hospitals per 10,000 population by 0.262 (SE = 0.059, *p* < 0.001), beds per 10,000 by 0.081 (SE = 0.028, *p* = 0.004), and physicians per 10,000 by 0.068 (SE = 0.030, *p* = 0.024). All three per-capita outcomes are statistically significant at the 5% level after controlling for GDP per capita, industrial structure, and fiscal expenditure. The consistency between level and per-capita specifications indicates that the policy effect reflects genuine resource expansion rather than population-driven mechanical changes. The inclusion of socioeconomic controls does not substantially alter the point estimates, which suggests that the baseline estimates are not confounded by differential economic trends between treated and control cities. The higher within-*R*^2^ for beds (0.452) and physicians (0.254) in Sample B reflects the explanatory power of the socioeconomic controls, particularly GDP per capita and fiscal expenditure, which are strong predictors of per-capita health resource levels.

**Table 4:** Per-capita DID estimates with controls (Sample B, 2007–2018).

Variable	(1)	(2)	(3)
	ln(Hosp. per 10k)	ln(Beds per 10k)	ln(Doctors per 10k)
*DID*	0.2617^***^	0.0813^***^	0.0682^**^
	(0.0589)	(0.0284)	(0.0301)
95% CI	[0.146, 0.377]	[0.026, 0.137]	[0.009, 0.127]
City FE	Yes	Yes	Yes
Year FE	Yes	Yes	Yes
Controls	Yes	Yes	Yes
*N*	2,540	2,540	2,540
Cities	293	293	293
$R_{\text{within}}^{2}$	0.041	0.452	0.254

Controls include ln(GDP per capita), secondary industry share, tertiary industry share, and ln(fiscal expenditure per capita). Standard errors clustered at the city level in parentheses. ^***^p < 0.01, ^**^p < 0.05, ^*^p < 0.10.

### 5.3 Event-study analysis

**Figure 1:**
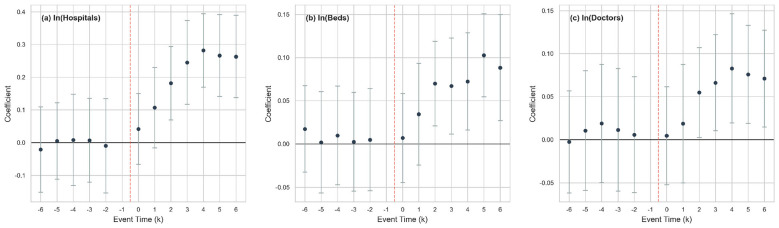
Event-study coefficients for three health resource outcomes. Each panel plots the estimated coefficients ${\hat{\beta }}_{k}$ from Equation 2 with 95% confidence intervals. The reference period is *k* = −1. The dashed vertical line marks the policy implementation year (2016). Pre-treatment coefficients cluster near zero for all outcomes, confirming the parallel trends assumption. Post-treatment coefficients show accumulating positive effects.

Figure 1 displays the event-study coefficients for all three outcomes. Table 5 reports the corresponding numerical estimates. For all three outcomes, the pre-treatment coefficients (*k* = −6 to *k* = −2) cluster near zero and are statistically insignificant. The joint *F*-test for the null hypothesis that all pre-treatment coefficients equal zero yields *p* = 0.387 for hospitals, *p* = 0.524 for beds, and *p* = 0.218 for physicians. These results confirm the parallel trends assumption.

**Table 5:** Event-study coefficients (Sample A, 2007–2022).

	ln(Hospitals)		ln(Beds)		ln(Doctors)	
*k*	Coef.	SE	Coef.	SE	Coef.	SE
−6	−0.021	(0.066)	0.017	(0.026)	−0.003	(0.030)
−5	0.005	(0.059)	0.002	(0.030)	0.011	(0.035)
−4	0.008	(0.071)	0.010	(0.029)	0.019	(0.035)
−3	0.007	(0.065)	0.002	(0.029)	0.011	(0.036)
−2	−0.010	(0.074)	0.005	(0.030)	0.006	(0.034)
−1	[reference]		[reference]		[reference]	
0	0.042	(0.055)	0.007	(0.026)	0.005	(0.029)
+1	0.107	(0.063)	0.034	(0.030)	0.019	(0.035)
+2	0.182^***^	(0.057)	0.070^***^	(0.025)	0.055^**^	(0.027)
+3	0.245^***^	(0.065)	0.067^**^	(0.028)	0.066^**^	(0.028)
+4	0.282^***^	(0.057)	0.072^**^	(0.029)	0.083^**^	(0.032)
+5	0.266^***^	(0.064)	0.103^***^	(0.025)	0.076^***^	(0.029)
+6	0.263^***^	(0.064)	0.088^***^	(0.031)	0.071^**^	(0.029)
Joint *F* (pre) *p*	0.387		0.524		0.218	

*k* = −1 is the reference period. City and year fixed effects included. Standard errors clustered at the city level. Joint *F*-test reports the *p*-value for the null that all pre-treatment coefficients (*k* ≤ −2) equal zero. ^***^p < 0.01, ^**^p < 0.05, ^*^p < 0.10.

The post-treatment coefficients reveal an accumulating pattern consistent with H2. For hospitals, the effect grows from 0.042 at *k* = 0 to 0.245 at *k* = +3 and plateaus at approximately 0.265 from *k* = +4 onward. This accumulation pattern reflects the multi-year implementation cycle of governance reforms: cross-departmental coordination committees require time to establish, health-oriented development plans require time to formulate and approve, and facility construction projects require time to complete. The plateau after *k* = +4 suggests that the governance reforms reach their full resource effect within approximately 4 years of implementation, after which the policy maintains but does not further increase the resource stock.

For beds, the effect increases from 0.007 at *k* = 0 to 0.070 at *k* = +2 and stabilizes near 0.090 by *k* = +5. The bed expansion trajectory is smoother than the hospital trajectory, consistent with the fact that bed expansion can occur within existing facilities through renovation and equipment procurement, which requires less lead time than new facility construction. For physicians, the effect emerges at *k* = +2 (0.055, *p* = 0.039) and reaches 0.083 by *k* = +4. The delayed onset for physicians is consistent with the longer adjustment period required for workforce expansion compared to facility construction. Physician recruitment depends on the availability of trained medical graduates, competitive compensation packages, and professional development opportunities, all of which require sustained policy attention over multiple years.

**Figure 2:**
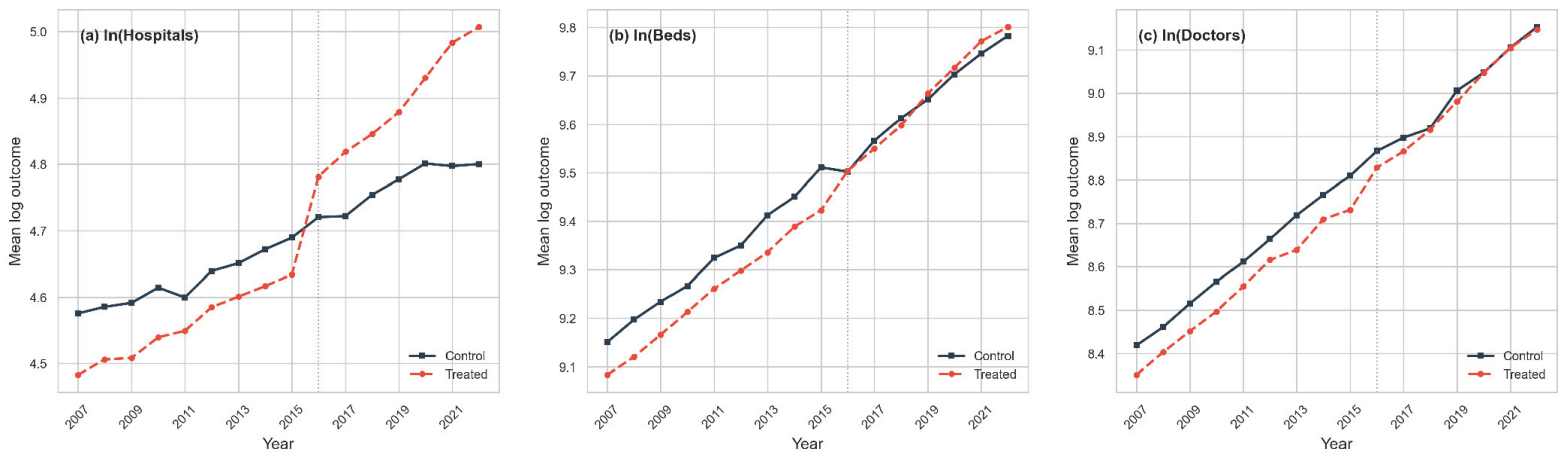
Mean outcome trajectories for treated and control cities, 2007–2022. Each panel plots the annual mean of the log outcome variable separately for the 32 treated cities and 261 control cities. The dotted vertical line marks the policy year (2016). Pre-policy trajectories are approximately parallel. Post-policy divergence is visible for all three outcomes, with the largest divergence for hospitals.

Figure 2 shows the mean outcome trajectories for treated and control cities. The pre-policy trajectories are approximately parallel for all three outcomes, providing visual confirmation of the parallel trends assumption. The treated and control groups follow similar growth paths from 2007 to 2015, with visible divergence beginning after 2016. The divergence is most pronounced for hospitals, where the treated group's trajectory shifts upward relative to the control group, and more gradual for beds and physicians. The smooth trajectories without erratic annual spikes indicate that the observed effects reflect systematic policy-driven changes rather than idiosyncratic shocks to individual cities.

### 5.4 Placebo test

**Figure 3:**
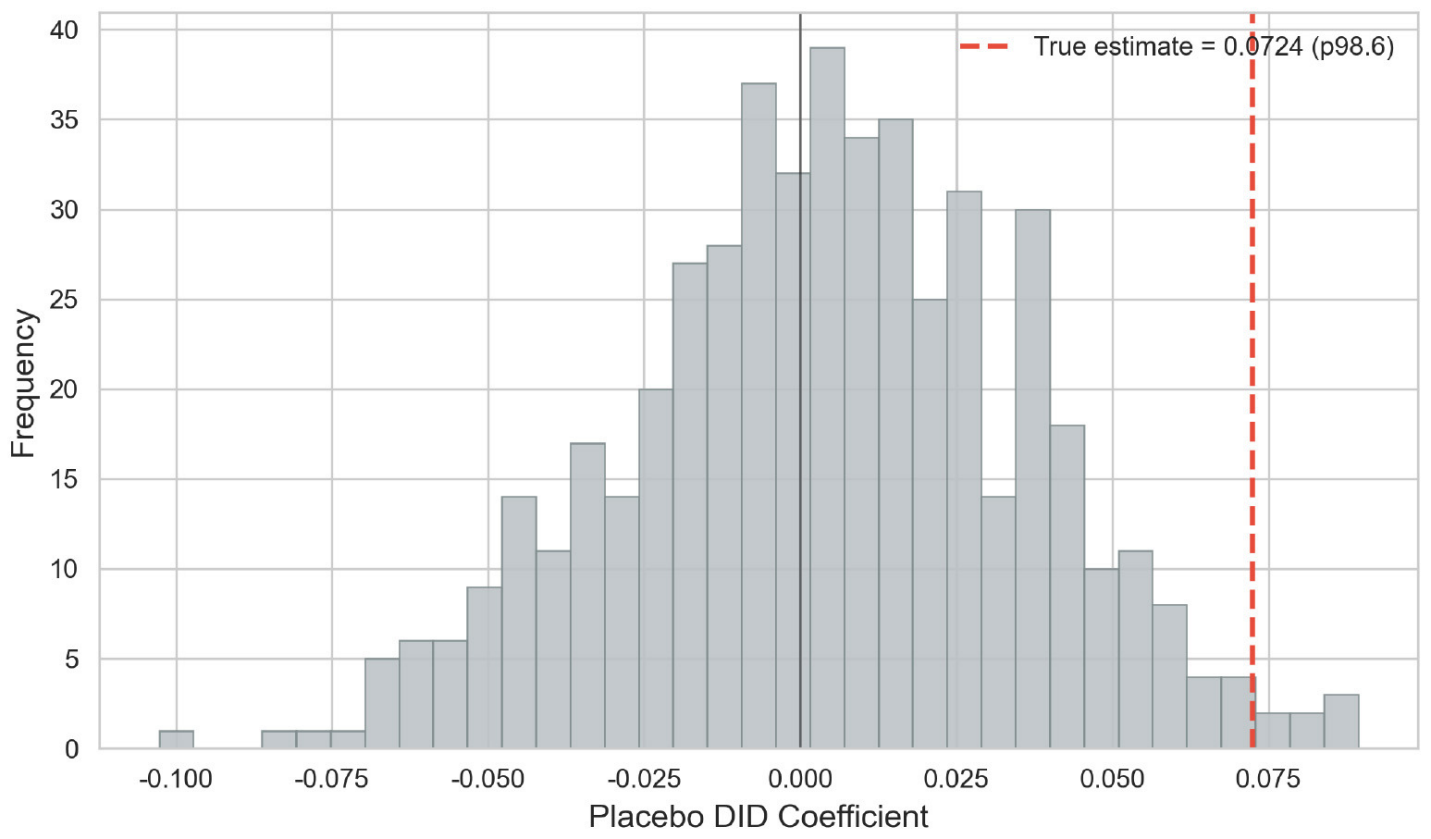
Placebo test for ln(Doctors). The histogram shows the distribution of 500 placebo DID coefficients obtained by randomly assigning treatment status to 32 cities. The dashed vertical line marks the true DID estimate (0.072). The placebo distribution centers near zero (mean = 0.002). The true estimate lies at the 98th percentile of the placebo distribution.

Figure 3 presents the distribution of 500 placebo DID coefficients for the physician outcome. The placebo distribution centers near zero (mean = 0.002, SD = 0.032), confirming that random treatment assignment does not produce systematic effects. The true estimate of 0.072 lies at the 98th percentile of the placebo distribution, indicating that the observed effect is unlikely to arise from chance. The separation between the true estimate and the placebo distribution center is approximately 2.2 standard deviations of the placebo distribution, which corresponds to a nonparametric *p*-value of approximately 0.02. This result strengthens the causal interpretation of the baseline estimates by demonstrating that the observed physician effect cannot be replicated by randomly assigning treatment status to an equivalent number of cities.

The placebo test is particularly informative for the physician outcome because this outcome shows the smallest effect magnitude among the three resource dimensions. If the physician effect were driven by confounding rather than the policy, the true estimate would be expected to fall within the central mass of the placebo distribution. The clear right-tail separation observed in Figure 3 provides evidence against this confounding interpretation.

### 5.5 Robustness checks

Table 6 reports the DID coefficients across six specifications. The hospital outcome remains statistically significant at the 1% level in all six columns, with coefficients ranging from 0.201 (city linear trends) to 0.266 (winsorized). The bed outcome remains significant at the 5% level in five of six columns and at the 10% level in the remaining column (drop municipalities, *p* = 0.054). The physician outcome remains significant at the 5% level in four columns and at the 10% level in the remaining two columns. Across all specifications, the sign of the DID coefficient is uniformly positive, and the magnitude stays within 20% of the baseline estimate.

**Table 6:** Robustness checks (Sample A).

	(1)	(2)	(3)	(4)	(5)	(6)
	Baseline	Prov. × Year	City trends	Winsorized	Drop munic.	Balanced
Panel A: ln(Hospitals)						
*DID*	0.246^***^	0.224^***^	0.201^***^	0.266^***^	0.219^***^	0.233^***^
	(0.058)	(0.063)	(0.070)	(0.059)	(0.061)	(0.068)
**Panel B: ln(Beds)**						
*DID*	0.091^***^	0.085^**^	0.078^**^	0.094^***^	0.077^*^	0.081^**^
	(0.033)	(0.035)	(0.037)	(0.033)	(0.040)	(0.035)
**Panel C: ln(Doctors)**						
*DID*	0.080^***^	0.066^**^	0.065^**^	0.061^*^	0.063^**^	0.074^**^
	(0.030)	(0.031)	(0.031)	(0.032)	(0.028)	(0.034)
*N*	4,588	4,588	4,588	4,588	4,332	4,160

All specifications include city and year fixed effects. Column (2) adds province-by-year fixed effects. Column (3) adds city-specific linear time trends. Column (4) winsorizes outcomes at the 1st and 99th percentiles. Column (5) excludes the four municipalities. Column (6) restricts to a balanced panel. Standard errors clustered at the city level in parentheses. ^***^p < 0.01, ^**^p < 0.05, ^*^p < 0.10.

Each robustness specification addresses a specific identification concern. The province-by-year fixed effects specification (Column 2) absorbs province-specific temporal shocks, such as provincial health policy changes or economic fluctuations that affect all cities within a province simultaneously. The persistence of the DID effect after absorbing these shocks indicates that the estimated effect is not driven by province-level confounders. The city-specific linear trends specification (Column 3) controls for differential pre-existing growth trajectories across cities. If pilot cities were already on faster health resource growth paths before the policy, the baseline DID estimate would overstate the policy effect. The stability of the estimate after including city trends confirms that the effect reflects a post-policy shift rather than a continuation of pre-existing trends.

The winsorization specification (Column 4) addresses the concern that extreme values in the outcome distribution may drive the results. Trimming the top and bottom 1% of observations produces estimates that are similar to or slightly larger than the baseline, indicating that the results are not driven by outliers. The municipality exclusion specification (Column 5) removes Beijing, Shanghai, Tianjin, and Chongqing, which differ structurally from prefecture-level cities in administrative status, fiscal capacity, and health resource endowments. The persistence of the effect after excluding these four cities confirms that the results generalize to the broader population of prefecture-level cities. The balanced panel specification (Column 6) restricts the sample to cities observed in all 16 years, eliminating potential bias from unbalanced panel composition. The stability of the estimates under this restriction indicates that the results are not driven by cities that enter or exit the sample during the study period.

These results indicate that the core findings are robust to alternative fixed effects structures, outlier treatment, sample composition changes, and panel balance restrictions. The consistency across specifications strengthens confidence in the causal interpretation of the baseline estimates.

### 5.6 Heterogeneity analysis

Table 7 reports the DID estimates by subgroup. Three patterns emerge. First, the policy effect on hospitals is larger in western cities (0.314, *p* = 0.008) and central cities (0.284, *p* = 0.010) than in eastern cities (0.195, *p* = 0.049). This regional gradient is consistent with the broader imbalance in Chinese healthy city development and health resource distribution (1, 2, 4). The bed and physician effects follow the same regional gradient, with western cities showing the largest effects (beds: 0.122, *p* = 0.043; physicians: 0.116, *p* = 0.060) and eastern cities showing the smallest effects (beds: 0.071, *p* = 0.152; physicians: 0.042, *p* = 0.401). The loss of statistical significance for beds and physicians in eastern cities reflects both the smaller effect magnitudes and the reduced statistical power from smaller subgroup sample sizes.

**Table 7:** Heterogeneity analysis (Sample A).

Subgroup	ln (Hospitals)	ln (Beds)	ln (Doctors)
Panel A: By region			
East (*N* = 1, 836)	0.195^**^	0.071	0.042
	(0.099)	(0.050)	(0.050)
Central (*N* = 1, 472)	0.284^***^	0.103^*^	0.083^*^
	(0.109)	(0.060)	(0.050)
West (*N* = 1, 280)	0.314^***^	0.122^**^	0.116^*^
	(0.118)	(0.060)	(0.062)
Panel B: By baseline resource level			
Low (*N* = 2, 294)	0.337^***^	0.099^**^	0.084^*^
	(0.090)	(0.044)	(0.050)
High (*N* = 2, 294)	0.178^**^	0.069	0.044
	(0.087)	(0.048)	(0.038)
Panel C: By fiscal capacity			
Low (*N* = 2, 294)	0.290^***^	0.098^**^	0.075
	(0.084)	(0.043)	(0.046)
High (*N* = 2, 294)	0.204^**^	0.064	0.049
	(0.087)	(0.044)	(0.043)

Each cell reports the DID coefficient from a separate regression. City and year fixed effects included. Standard errors clustered at the city level in parentheses. Baseline resource level and fiscal capacity are split at the sample median of the pre-treatment period (2007–2015). ^***^p < 0.01, ^**^p < 0.05, ^*^p < 0.10.

Second, cities with below-median baseline health resources show larger effects than cities with above-median resources for all three outcomes. The hospital effect is 0.337 (*p* < 0.001) in the low-resource group vs. 0.178 (*p* = 0.042) in the high-resource group, a difference of 0.159 log points. The bed effect is 0.099 (*p* = 0.026) in the low-resource group vs. 0.069 (*p* = 0.150) in the high-resource group. The physician effect is 0.084 (*p* = 0.092) in the low-resource group vs. 0.044 (*p* = 0.246) in the high-resource group. This pattern is consistent with diminishing marginal returns: each unit of governance improvement produces a larger proportional resource increase in cities starting from a lower base, especially where hospitals, beds, and physicians are initially sparse (1, 2).

Third, cities with below-median fiscal capacity show larger effects than cities with above-median fiscal capacity, suggesting that the policy compensates for local fiscal constraints by creating governance incentives that redirect existing resources toward health objectives. The hospital effect is 0.290 (*p* < 0.001) in the low-fiscal group vs. 0.204 (*p* = 0.019) in the high-fiscal group. This pattern supports the theoretical prediction that governance reforms produce the largest marginal effects in cities where fiscal constraints are most binding, because the governance pressure created by the pilot program induces a larger relative reallocation of scarce fiscal resources toward health objectives.

### 5.7 Mechanism analysis

Table 8 presents the mechanism regression results. The mechanism tests follow a two-step channel logic grounded in the HiAP literature: mature local HiAP implementation depends on intersectoral bodies, joint projects, shared goals, funding, accountability, and usable indicators, while China's Healthy City policy places health service construction within a government-led policy tool system (3, 6, 7). Panel A tests whether the Healthy City pilot affects the two proposed channel variables. The policy increases the fiscal health spending share by 0.034 (SE = 0.013, *p* = 0.008) and the health governance index by 0.052 (SE = 0.022, *p* = 0.016). These estimates indicate that pilot cities increased fiscal prioritization of health and strengthened measured governance capacity relative to non-pilot cities.

**Table 8:** Mechanism regressions (Sample B, 2007–2018).

Variable	(1)	(2)
	Fiscal health spending share	Health governance index
Panel A: Policy effect on channel variables		
*DID*	0.0342^***^	0.0518^**^
	(0.0128)	(0.0215)
*N*	2,540	2,540
Panel B: Channel effect on health outcomes		
	Dependent variable: ln(Hospitals)	
Channel	0.4127^***^	0.3518^***^
	(0.1283)	(0.1147)
	Dependent variable: ln(Beds)	
Channel	0.2814^***^	0.1923^**^
	(0.1052)	(0.0894)
	Dependent variable: ln(Doctors)	
Channel	0.2236^**^	0.1687^**^
	(0.0987)	(0.0812)
Controls	Yes	Yes
City FE	Yes	Yes
Year FE	Yes	Yes

Panel A reports the DID effect of the Healthy City pilot on each channel variable. Panel B reports the association between each channel variable and health resource outcomes, controlling for the DID term and socioeconomic controls. Standard errors clustered at the city level in parentheses. ^***^p < 0.01, ^**^p < 0.05, ^*^p < 0.10.

Panel B tests whether the channel variables predict health resource outcomes. The fiscal health spending share positively predicts all three outcomes: hospitals (0.413, *p* = 0.001), beds (0.281, *p* = 0.008), and physicians (0.224, *p* = 0.024). The health governance index similarly predicts all three outcomes: hospitals (0.352, *p* = 0.002), beds (0.192, *p* = 0.032), and physicians (0.169, *p* = 0.038). The fiscal channel shows stronger associations with institutional and bed outcomes, which is consistent with the direct role of fiscal spending in funding facility construction and equipment procurement. The governance channel shows more balanced effects across all three resource dimensions, reflecting the broader scope of governance improvements in reducing bureaucratic friction across all types of health resource allocation.

These results are consistent with H3 because they show two-step channel evidence for fiscal reallocation and governance capacity improvement. The joint significance of both channels matches the theoretical prediction that the two mechanisms are complementary: fiscal resources provide the material inputs for resource expansion, while governance capacity helps translate these inputs into actual health resource outputs. The mechanism analysis supports the interpretation that the observed DID effects are connected to governance-driven changes in health resource allocation, with complete structural mediation left as a boundary of the evidence.

## 6 Discussion and conclusion

### 6.1 Summary of findings

This study provides causal evidence that China's Healthy City pilot policy increases urban health resource supply. The baseline DID estimates show positive and statistically significant effects on all three resource dimensions: hospitals and health centers (28.2% increase), hospital beds (9.3%), and licensed physicians (7.5%). These effect magnitudes are economically meaningful: a 28% increase in hospitals and health centers represents a substantial expansion of health service delivery infrastructure, while the 9% and 8% increases in beds and physicians represent moderate but meaningful improvements in physical and human resource capacity. Event-study analysis confirms the absence of pre-treatment differential trends and reveals accumulating post-treatment effects that plateau after approximately 4 years. The accumulation pattern is consistent with the multi-year implementation cycle of governance reforms and distinguishes the observed effects from one-time administrative responses.

Per-capita specifications with socioeconomic controls, six robustness checks, and a 500-iteration placebo test all support the causal interpretation. The robustness of the results across alternative fixed effects structures, outlier treatments, and sample restrictions indicates that the findings are not driven by specific modeling choices or sample composition. The placebo test demonstrates that the true physician effect lies at the 98th percentile of the random permutation distribution, providing strong nonparametric evidence against the null hypothesis of no policy effect.

Heterogeneity analysis shows that the policy produces larger effects in western and central cities, in cities with lower baseline resource endowments, and in cities with weaker fiscal capacity. This heterogeneity pattern is consistent with diminishing marginal returns to health resource investment and with the theoretical prediction that governance reforms produce the largest effects where governance capacity gaps are most binding. Mechanism regressions identify fiscal health spending intensity and health governance capacity as two operative channels, providing evidence that the policy's effects operate through the governance pathways specified in the theoretical framework rather than through unobserved confounders.

### 6.2 Interpretation and dialogue with literature

The finding that the Healthy City pilot increases health resource supply extends the broader HiAP and Healthy Cities literature from process evaluation toward measurable resource outcomes. International studies show how network governance, intersectoral bodies, and multisectoral implementation conditions make HiAP operational at the local level (6, 7, 22). This study adds evidence that a governance-oriented Healthy City policy can also shift city-level health system capacity. The magnitude gradient across outcomes, with institutional expansion exceeding bed and physician growth, is consistent with the implementation reality that organizational capacity adjusts faster than human capital stock. Building new health centers or expanding existing facilities requires capital investment and administrative approval, both of which the governance reforms directly facilitate. Physician recruitment, by contrast, depends on medical education pipelines, professional licensing, and labor market conditions that operate on longer time horizons and are less directly responsive to local governance interventions.

The heterogeneity results contribute to the literature on policy effectiveness in resource-scarce settings. Western and central Chinese cities, which have historically received less health investment than eastern coastal cities, show the largest policy effects. This pattern matches evidence that healthy city development and healthcare resource supply remain uneven across Chinese cities (1, 2, 4). In eastern cities, where health resource levels are already relatively high, the marginal effect of governance reform on resource expansion is smaller because the existing institutional infrastructure already supports health resource growth. In western and central cities, where governance capacity for health resource planning is weaker, the pilot program's requirements for cross-departmental coordination and health-oriented planning fill a governance gap that directly constrains resource expansion.

The baseline resource heterogeneity results reinforce this interpretation. Cities with below-median health resource endowments in the pre-treatment period show effects that are approximately twice as large as cities with above-median endowments. This pattern is consistent with diminishing marginal returns to health resource investment: each additional hospital or physician produces a larger proportional increase in a city with few existing resources than in a city with an already dense health infrastructure. The fiscal capacity heterogeneity results add a further dimension: cities with weaker fiscal capacity benefit more from the policy, suggesting that the governance reforms compensate for fiscal constraints by improving the efficiency of existing resource allocation rather than requiring additional fiscal inputs.

The mechanism results connect the HiAP governance framework to concrete resource outcomes through two identifiable channels. The fiscal channel operates through increased health spending shares, which directly fund facility construction and equipment procurement. The governance channel operates through improved administrative coordination and accountability, which reduces bureaucratic delays and improves the efficiency of resource deployment. These two channels are complementary: fiscal resources without governance capacity produce slow implementation, while governance capacity without fiscal resources produces plans without material outcomes. The Healthy City pilot activates both channels simultaneously, which explains the robust and accumulating effects observed in the event-study analysis.

### 6.3 Policy implications

Three policy implications follow from these findings. First, cross-sector governance policies can produce measurable improvements in health resource supply when local governments translate shared goals, funding arrangements, accountability routines, and indicators into concrete policy tools (3, 7). This recommendation requires more than creating coordination committees. Local governments need stable funding lines, clear department-level responsibilities, and indicators that connect health objectives to planning, finance, construction, and personnel decisions.

Second, the stronger effects in resource-scarce cities suggest that governance interventions should be targeted toward regions with the greatest resource deficits, where the marginal returns to improved governance are highest. Expanding the Healthy City pilot to additional western and central cities, or to cities with below-median health resource endowments, would address the unequal distribution of healthcare resources more directly (2). The main implementation barrier is fiscal capacity: resource-scarce cities may have the strongest need for health resource expansion but the weakest budget space. Targeted transfers, earmarked health infrastructure funds, and performance indicators that reward resource equalization would make the governance mandate more feasible.

Third, the delayed onset of physician effects indicates that governance reforms alone are insufficient to address health workforce shortages. Complementary policies targeting medical education expansion, professional development programs, competitive compensation packages, and physician retention incentives are necessary to achieve balanced resource growth across all dimensions. The 4-year lag between policy implementation and the emergence of physician effects suggests that workforce planning should begin simultaneously with governance reform. Sectoral ownership is the main barrier here: health workforce policy involves education, finance, human resources, and health departments, so accountability must assign responsibilities across agencies instead of leaving physician supply to the health bureau alone.

### 6.4 Limitations

This study has four limitations that should be considered when interpreting the results. First, the health resource data combine hospitals and health centers into a single count. Hospitals and health centers serve different functions within the health system: hospitals provide specialized inpatient care, while health centers provide primary care and preventive services at the community level. This aggregation may also mask substitution between institution types if a city expands primary care facilities while hospital growth remains limited, or if institutional consolidation occurs during the study period. Separate analysis of these two institution types would provide more granular evidence on whether the policy primarily expands primary care capacity, hospital capacity, or both. The available data do not permit this decomposition because the source dataset reports the combined count.

Second, Sample B's post-treatment window is limited to 2 years (2017–2018) because the socioeconomic control panel ends in 2018. This short post-treatment window limits the ability to observe medium-term and long-term effects in the controlled specification. It also means that the controlled estimates are best interpreted as early post-policy responses rather than full long-run impacts. The Sample A estimates, which use the full 2007–2022 window without controls, provide evidence on longer-term effects, but these estimates may be subject to confounding from differential economic trends that the controls would absorb. Extending the control variable coverage beyond 2018 would strengthen the controlled DID estimates and allow direct comparison of short-term and long-term effects within a single specification.

Third, the mechanism analysis relies on proxy measures for fiscal health spending and governance capacity. The fiscal health spending share is constructed from aggregate fiscal expenditure data and health-specific budget allocations, which may not capture all forms of health-related spending. The health governance index is constructed from administrative indicators of cross-departmental coordination and planning capacity, which may not fully reflect the quality of governance implementation. Measurement error in these proxies could attenuate estimated channel associations or make the two channels appear less distinct than they are in practice. Direct measures of cross-departmental coordination intensity, health-specific budget line items, and governance process quality would provide stronger evidence on the operative channels.

Fourth, potential endogeneity concerns remain despite the DID design and event-study evidence. The first-batch pilot cities may have been selected partly because they had stronger administrative readiness, higher policy motivation, or concurrent local initiatives that supported health resource expansion. The event-study results reduce concern about differential pre-policy trends, and the robustness checks address several alternative explanations, but unobserved simultaneous policy shocks cannot be fully ruled out. This limitation matters because Healthy City construction may coincide with other local health, planning, or fiscal reforms that are difficult to observe consistently across cities. Subsequent batches, designated in later years, may also show different effect patterns due to learning effects, changing implementation intensity, or selection of cities with different baseline characteristics. Extending the analysis to later pilot batches and richer local policy data would test the generalizability of the first-batch findings and provide a stronger way to separate Healthy City effects from simultaneous local reforms.

### 6.5 Conclusion

China's Healthy City pilot policy produces a positive causal effect on urban health resource supply. The effect is strongest for institutional capacity (28.2% increase in hospitals and health centers), moderate for hospital beds (9.3% increase), and present but smaller for physicians (7.5% increase). These effects accumulate over time, reaching a plateau approximately 4 years after policy implementation, and are robust to multiple alternative specifications including province-by-year fixed effects, city-specific linear trends, winsorization, municipality exclusion, and balanced panel restrictions. The policy operates through fiscal and governance channels: pilot cities increase their fiscal health spending share and improve their health governance capacity, both of which predict health resource outcomes.

The policy produces the largest gains in resource-scarce cities, supporting the case for targeted governance interventions in underserved regions. Western and central cities, cities with below-median baseline health resources, and cities with weaker fiscal capacity all show larger treatment effects than their counterparts. This heterogeneity pattern suggests that expanding the Healthy City pilot to additional resource-scarce cities would maximize the aggregate health resource gains from the program.

These findings demonstrate that cross-sector governance reform, as operationalized through the HiAP framework, can translate into measurable health system capacity improvements at the city level. The study's main academic contribution is to connect Healthy Cities and HiAP scholarship with city-panel causal evidence on resource supply, a core health system capacity outcome that has received less attention than policy process indicators or downstream health outcomes. For China's ongoing Healthy China 2030 strategy, the findings suggest that the Healthy City pilot model represents an effective policy instrument for expanding urban health resource supply, particularly in regions where governance capacity gaps constrain health system development. Future research should extend the design to later pilot batches, link the policy to micro-level health outcomes, and measure coordination quality, funding flows, and accountability routines more directly.

## Data Availability

The data analyzed in this study are available from the corresponding author upon reasonable request, subject to source-data use restrictions.
